# Lipid lowering by hydroalcoholic extracts of *Amaranthus Caudatus L*. induces regression of rabbits atherosclerotic lesions

**DOI:** 10.1186/1476-511X-10-89

**Published:** 2011-05-28

**Authors:** Najmeh Kabiri, Sedigheh Asgary, Mahbubeh Setorki

**Affiliations:** 1 Department of Biology, Faculty of Sciences, Isfahan University, Isfahan, Iran; 2 Isfahan Cardiovascular Research Center, Applied Physiology Research Center, Isfahan University of Medical Sciences, Isfahan, Iran; 3 Department of Biology, Izeh Branch, Islamic Azad University, Izeh, Iran

## Abstract

**Background:**

The antihypercholesterolemic and antiatherogenic effect of hydroalcoholic extracts of *Amaranthus caudatus L*(A. *caudatus*). on regression of atherosclerosis in experimental rabbits maintained on a high cholesterol diet.

**Methods:**

Twenty five rabbits were randomly divided into five groups of five each and treated 75 days as follows: Group I: normal diet(ND), Group II: Hypercholesterolemic diet (HCD) for 45 days; Group III: Hypercholesterolemic diet (HCD) for 75 days, Group IV and V: HCD for 45 days and then normal diet and normal diet + A. *caudatus*(150 mg·kg day) respectively for an additional 30 days(regression period). Blood samples were collected before (0 time) and after 45 days and 75 days of experimental diets for measurement of biochemical factors. The aortas were removed at the end of the study for assessment of atherosclerotic plaques.

**Results:**

In regression period dietary use of A. *caudatus *in group V significantly decreased total cholesterol, LDL-cholesterol, malondialdehyde, C-reactive protein while apolipoproteinA and HDL- cholesterol was significantly increased compared to group IV. The atherosclerotic area was significantly decreased in group V. Whereas, the animals that in regression period received only normal diet showed no regression but rather progression of atherosclerosis.

**Conclusion:**

These results thus suggest that hydroalcoholic extracts of A. *caudatus *can reduce risk factors and cause regression of fatty lesons in aorta.

## Background

Atherosclerosis is a dynamic and reversible process [1]. In animals fed a cholesterol-rich diet, lowering of plasma cholesterol levels promotes the regression of atherosclerotic lesions [1-3]. Evidence for atherosclerosis regression in humans has also been reported [4-6]. Although plasma lipid lowering is a major driving force, the mechanisms that promote lesion regression and stabilization are not clear [7]. Recent reports indicate that the reversal or regression of lesions can be achieved by aggressive lipid lowering or drug treatment [8,9]. Hypercholesterolemia induces oxidative stress, which is known to have adverse effects on the integrity of cells [10]. Antioxidants and hypolipidemic agents suppress the development of hypercholesterolemic atherosclerosis and induce regression of atherosclerosis. Suppresses the development of atherosclerosis is associated with decreases in oxidative stress and serum lipids [11-13].

*Amaranthus Caudatus L*.( *A.Caudatus*) which is synonym with *Amaranthus Paniculatus L*. was used in some studies. Grain and leaves of *Amaranthus *are utilized as food for human beings as well as for animals [14] and their nutritional value have been extensively studied [15]. *Amaranthus *leaves are an excellent source of protein, fiber, squalene, anthocyanins and tocotrienols [16-18]. Squalene is an intermediate in cholesterol biosynthesis and is found in humans under the skin and inside the adipose tissue [17]. The physiological effects of dietary flavonols are of current interest due to their *in vitro *anti-oxidative and anti-inflammatory activities [18]. It was recently shown positive effect of *A. caudatus *extract to decrease of cholesterol level, reduced serum lipid levels to atheromatous plaque formation [19,20]. Qureshi et al. (1991), shows that *A. caudatus *extracts contains tocotrienol and tocopherol [21]. Recently, it has shown that these two substance regulate cholesterol metabolism [22].

This study assessed the ability of *Amaranthus Caudatus L*. to reduce atheromatous plaque formation and also its ability to regress atheromatous lesions in hypercholesterolemic rabbits by analysing biochemical markers such as total cholesterol, triglyceride and LDL-cholesterol, apolipoproteinB, malondialdehyde inflammatory markers such as C-reactive protein (CRP) and lesion in the aorta by histochemical analysis.

## Material and Methods

### Collection of plant material and extraction

*A. caudatus *was collected from Isfahan Natural Resource Institute. They were identified by Dr. Lili Ghaemmaghami and a voucher specimen was deposited at the Herbarium of the Department of Biology, Faculty of Science and Isfahan University (voucher no. 13648). Aerial parts (stems, leaves and flowers) were dried for 10 days at room temperature it was extracted with 96% ethanol for 72 hours and then filtered, and concentrated by vacuum distillation. Solvent was evaporated under vacuum and crude extract was obtained as a dark reddish colour and kept in dark glass bottles at 4°C until use [23].

### Flavonoid and anthocyanines measurement

Total flavonoids content was measured at 424 nm, [24] and anthocyanins at 535 nm [25] using spectrophotometeric method.

### Animals and treatment

Twenty five male New Zealand white rabbits with average body weight of 1.5-2 kg were purchased from Razi Institute, Teheran, Iran. The animals were housed in steel cages individually in a temperature and light controlled room for 2 weeks, and provided with Super Fosskorn Normal Rabbit Chow, purchased from Pasteur Institute of Iran.

#### Regression studies

The rabbits were divided into five groups of five rabbits each and for 75 days as follows.

▪ Group І: Fed with 100 g Normal diet daily (normal group). (75 days).

▪ Group ΙΙ: Normal diet + cholesterol suspended in olive oil and added to the diet (1% of food content daily)Which is considered to be a high cholesterol diet(HCD) throughout the experiment (45 days).

▪ Group III: Normal diet + cholesterol suspended in olive oil and added to the diet (1% of food content daily)which is considered to be a high cholesterol diet(HCD) throughout the experiment (75 days).

**For regression studies**, 10 rabbits were a 1% cholesterol diet for 45 days and the rabbits were divided into two groups:

▪ Group IV: Normal diet for 30 days.

**Figure 1:**
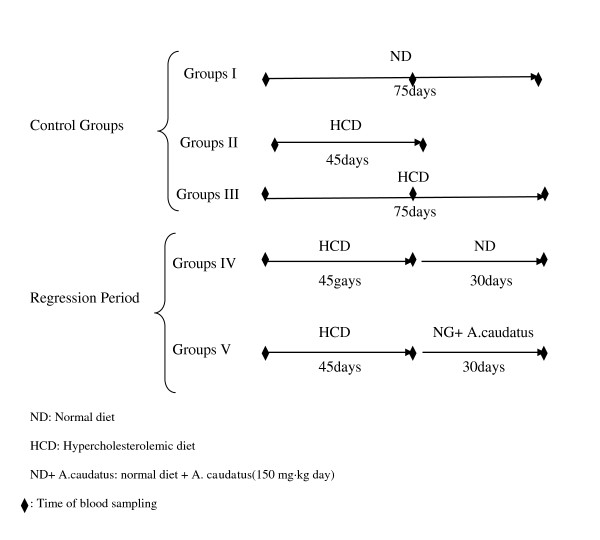
**Scheme of the experimental regression period showing the days**.

▪ Group V: Normal diet + *A. caudatus *extract(150 mg kg body weight daily) for 30 days. The study design is summarized in Figure 1.

Isfahan Cardiovascular Research Center Ethics Committee which is a member of office for human research protections, US department of health and human services, approved the present study, and the animals were handled according to guidelines of Isfahan University of Medical Sciences for Laboratory Animal Sciences for the care and use of laboratory animals.

### Measuring the biochemical factors

Blood samples (from marginal ear vein) after 12 hours of fasting were collected before time 0, 45th & 75th days. The plasma was obtained by centrifuging the blood samples at 2000 rpm for 15 min Total cholesterol(cho), Triglyceride(TG) and LDL-cholesterol (LDL-C) and HDL-cholesterol (HDL-C) were measured using special kits (DiaSys, Germany) which utilized the colorimetric method, in an autoanalyzer (Hitachi autoanalyzer, Hitachi Co., Tokyo). Concentrations of apolipoproteinA (apoA) and apolipoproteinB(apoB) were also measured using special kits (DiaSys, Germany) in an autoanalyzer (Hitachi autoanalyzer, Hitachi Co., Tokyo) according to the turbidimetric method; C-reactive protein (CRP) was also measured by rabbit CRP ELISA (Rapidbio, USA). We measured Ox-LDL by rabbit Ox-LDL ELISA (Rapidbio, USA). Malondialdehyde (MDA) was estimated by the double heating method of Draper and Hadley. The principle of the method is the spectrophotometric measurement of the color generated by the reaction of thiobarbituric acid with MDA.

All these factors were measured at the beginning, 30th and 60th day except for Ox-LDL which was checked only at 60th day. Atherosclerosis index was calculated according to the following formula: AI = LDL-C/HDL-C [26].

On the 75th day all the groups of animals, were sacrificed by rapid intracardiac pentobarbital injection for histopathological analyze.

### Histopathological analysis of liver and aorta

At the end of experiment, the animals were killed, the aorta were removed and tissue sections (5 μm) aorta fixed by immersion at room temperature in 10% formalin solution stained for fat deposits. For the histological examinations, paraffin-embedded tissue section of aorta were stained with hematoxylin-eosin (H&E). The tissue samples were then examined and photographed under a light microscope for observation of structural abnormality. Chekanov scale was used for grading of atherosclerotic plaques and the results were determined on a scale of 1-4 in relation to the thickness of media layer as follows:

▪ Grade 1. Plaque less than half as thick as the media with some form of endothelial dysfunction

▪ Grade 2: Plaque at least half as thick as media with accumulation of intracellular lipid, macrophages, and smooth muscle cells.

▪ Grade 3: Plaque as thick as the media with an abundance of macrophages, smooth muscle cells, and connective tissue.

▪ Grade 4: Plaque thicker than the media with a large intracellular intimal lipid core and inflammatory cell infiltration [27].

All histopathological evaluations were done by a pathologist blinded to the experimental design.

### Statistical analysis

All values were expressed as mean ± SD Significant differences among the groups were determined by one-way ANOVA using the SPSS 13.0 software package program. Values of p < 0.05 were taken as statistically significant.

## Results

### Amount of flavenoids and anthocyanins

Each 100 g powder of A. caudatus results in 3.8 ± 0.029 g extract powder. The amount of total flavonoids based on hyperoside and anthocyanins in 100 g of *A. caudatus *extract is 0.379 ± 0.02 g and 24.1 ± 1.29 mg respectively.

### Changes in biochemical parameters level in serum

-At the onset, no significance was found between the mean values among the study groups. Keeping the rabbits on high cholesterol diet significantly increased Cho, TG, LDL-C, HDL-C, apoB, AI, CRP, MDA, OX-LDL and apoA in Groups II, III, ΙV and V on the 45^th ^days, as compared to Group Ι, However after the 75^th ^days in Groups III as compared to Group I(Table 1).

**Table 1:** Effects of hydroalcoholic extract of *A. caudatus *on Selective biochemical parameters in rabbits fed with high cholesterol diets on 0, 45^th ^and 75^th ^days

Biochemical parameters	Duration (days)	GroupI	GroupΙΙ	GroupIII	GroupIV	GroupV
	0	41.4 ± 8.443933	47.6 ± 7.436397	46.8 ± 7.463243	45.4 ± 8.792042	45.8 ± 6.723095
	
	45	46.8 ± 7.463243	898.2 ± 84.3872	664 ± 192.3382	786.2 ± 180.7836	876.8 ± 100.3927
	
Cholesterol(m g/dl)	75	47.6 ± 7.436397	-------------	1053.2 ± 34.76636	1020.8 ± 72.58237	575 ± 34.12477ba

	0	45.267 ± 11.58879	55.8 ± 6.797058	58.2 ± 7.823043	44.2 ± 12.39758	59 ± 10.65364
	
	45	45.267 ± 12.39758	166.2 ± 30.32656	109.4 ± 20.59854	156.2 ± 36.46505	131.6 ± 7.700649
	
Triglyceride (mg/dl)	75	46.233 ± 1.16301	-------------	238.4 ± 55.13892	195.8 ± 54.29273	85.6 ± 7.700649

	0	16.42 ± 1.99925	17.42 ± 1.485598	15.086 ± 1.117578	16.96 ± 1.7358	16.98 ± 1.01341
	
	45	17.1 ± 1.604681	837.2 ± 65.78146	839.2 ± 46.21904	813.2 ± 73.41117	660.62 ± 361.4526
	
LDL-cholesterol mg/dl)	75	17.38 ± 1.070981	--------------	952.98 ± 50.06078	694.88 ± 45.25231b	426.8 ± 36.0236b

	0	23.4 ± 9.607289	16.8 ± 3.114482	18.6 ± 2.607681	17.4 ± 3.130495	19.4 ± 6.107373
	
	45	20.8 ± 5.630275	61.6 ± 12.05404	56.8 ± 10.56882	82 ± 14.91643	70.8 ± 10.84896
	
HDL- cholesterol (mg/dl)	75	19 ± 5.43139	--------------	81.2 ± 16.42255	86.4 ± 17.85497	134.2 ± 11.14451ba

	0	8.2 ± 1.48324	10.2 ± 4.91935	13.6 ± 4.037326	14.2 ± 3.563706	14.4 ± 3.781534
	
	45	14 ± 4.52769	34.8 ± 10.32957	30 ± 9.082951	27 ± 5.522681	28.8 ± 4.658326
	
apolipoprotein B (mg/dl)	75	9.8667 ± 1.30384	-------------	58.2 ± 11.43241	49.2 ± 11.64903	41 ± 8.602325

	0	43 ± 9.823441	42.2 ± 9.364828	43.6 ± 11.90798	41.8 ± 8.757854	41.4 ± 12.83745
	
	45	47.8 ± 5.215362	27.2 ± 7.362065	27.6 ± 7.924645	28.8 ± 6.648308	29.6 ± 6.308724
	
apolipoprotein A (mg/dl)	75	34.8 ± 8.288546	--------------	22.4 ± 7.765307	21 ± 9.66954	52.4 ± 8.961027ba

	0	0.3 ± 0.141421	0.16 ± 0.089443	0.2 ± 0.141421	0.2 ± 0.141421	0.2 ± 0.1
	
	45	0.2 ± 1.604681	0.7 ± 0.070711	0.68 ± 0.164317	0.64 ± 0.151658	0.7 ± 0.070711
	
de Malondialdehy (mol/l)	75	0.22 ± 0.130384	--------------	1.7 ± 0.234521	0.92 ± 0.204939b	0.35 ± 0.070711ba

	0	3.02 ± 0.141421	3.66 ± 1.934683	2.26 ± 1.062073	2.66 ± 1.825651	2.32 ± 1.634625
	
C-reactive protein (mg/l)	45	2.96 ± 1.564609	12.82 ± 3.094673	11.36 ± 3.706481	14.08 ± 2.259867	12.46 ± 3.304996
	
	75	2.58 ± 1.88202	--------------	23.4 ± 3.361547	8.9 ± 0.514782b	5.1 ± 1.031988ba

	0	15.12 ± 1.41492	15.26 ± 1.209545	15.22 ± 1.243785	15.72 ± 2.667771	14.46 ± 1.209545
	
	45	16.22 ± 2.615722	23.32 ± 2.963444	23.32 ± 3.013636	25.72 ± 4.130617	26.12 ± 3.62519
	
OX- LDL(ng/ml)	75	15.5 ± 2.382226	--------------	35.12 ± 5.157713	25.54 ± 3.72599	18.82 ± 1.411382b

	0	42.358 ± 8.124376	46.554 ± 7.265138	45.284 ± 8.458223	45.862 ± 12.62378	44.846 ± 6.728988
	
	45	45.926 ± 7.531051	884.204 ± 83.63083	729.088 ± 148.0296	738.548 ± 148.253	867.5016 ± 102.6377
	
AI	75	46.642 ± 7.538247	--------------	1040.96 ± 33.85897	1011.03 ± 26.33166	551.062 ± 71.06503b

Group І: Fed with Normal diet (control group) (75^th ^days)

Group ΙΙ: Normal diet + cholesterol (45^th ^days)

Group ΙIΙ: Normal diet + cholesterol (75^th ^days)

Group ΙV: Normal diet + cholesterol for 45^th ^days and Normal diet for 30^th ^days

Group V: Normal diet + cholesterol for 45^th ^days and Normal diet + *A. caudatus *for 30^th ^days

b Significant difference between Group III with Groups IV Group V. (p < 0.05)

c Significant difference between Group IV with Group V. (p < 0.05)

Cho: total cholesterol, TG:Triglyceride, LDL-C: LDL-cholesterol, HDL-C: HDL-cholesterol, ApoB:apolipoproteinB, ApoA:polipoproteinA, MDA: malondialdehyde,

CRP:C-reactive protein, AI: atherosclerosis index

In regression period dietary use of *A. caudatus *in Group V significantly decreased Cho, LDL-C, CRP, MDA, OX-LDL and AI in compared Group III on the 75^th ^days, whereas in this period in the group fed Normal diet (GroupIV) significantly decreased LDL-C, CRP and MDA in compared Group III on the 75^th ^days(Table 1).

During the regression, in the group fed with *A. caudatus *in addition to Normal diet (Group V) the levels of cho, LDL-C, MDA and CRP were found to be significantly decreased, whereas TG, apoB, AI and OX-LDL nonsignificantly decreased compared to the rabbits fed normal diet (Group IV), on the other hand, this diet the levels of apoA and HDL-C significantly increased in compared Group IV (Table 1).

### Aortic of atherosclerotic lesions

**Figure 2:**
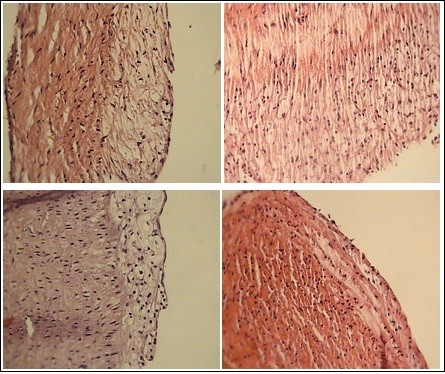
**Histology of aorta and grade of atherosclerotic plaque in studied groups**. a: Normal diet + cholesterol (45^th ^days) b: Normal diet + cholesterol (75^th ^days) c: Normal diet + cholesterol for 45^th ^days and Normal diet for 30^th ^days d: Normal diet + cholesterol for 45^th ^days and Normal diet + *A. caudatus *for 30^th ^days.

The aorta atherosclerotic lesions of GroupI rabbits showed normal histology. However aortic of atherosclerosis of GroupII (45^th ^days) and GroupIII (75^th ^days) respectiv (1.203 ± 0.34356) and (2.76 ± 0.360555). The ratio of the atherosclerotic lesions area of the GroupIV(1.266 ± 0.416333) and GroupV (086 ± 0.503322) was significantly smaller (p < 0.05) than that the GroupIII(table 2). The was a nonsignificant reduction in the aorta atherosclerotic rabbits fed on normal diet + *A. caudatus *for 30^th ^days(GroupV) compared with rabbits fed on normal diet for 30^th ^days(GroupIV), indicating that *A. caudatus *accelerated the regression of atherosclerotic lesions (Figure 2).

**Table 2:** Comparison of atheroma plaque in aortic cuts of four groups of rabbits fed with high cholesterol diets

Groups	Plaque thickness	Plaque stage	Plaque thickness to media thickness
GroupII	1.2 ± 0.34356	1	More than half of media thickness

Group III	2.76 ± 0.360555	2	Plaque at least half as thick as media

Group IV	1.26 ± 0.416333^**a**^	1	More than half of media thickness

Group V	086 ± 0.503322 ^**a**^	1	More than half of media thickness

Group ΙΙ: Standard diet + cholesterol (75^th ^days)

Group ΙΙI: Standard diet + cholesterol (45^th ^days)

Group IV: Standard diet + cholesterol for 45^th ^days and Standard diet for 30^th ^days

Group V: Standard diet + cholesterol for 45^th ^days and Standard diet + *A. caudatus *for 30^th ^days

aSignificant difference between Group V and Group IV with GroupII. (p < 0.05)

## Discussion

The results presented above indicate that *Amaranthus*, has significant antihyperlipidemic and antiatherogenic effects and promoted the regression of atheromatous lesions. This was evidenced by decrease in the extent of accumulation of cholesterol and triglycerides in serum and aorta of rabbits to slow lipid peroxidation process (lower Ox-LDL and MDA), and finally to enhance the inflammatory response of the endothelial cells and accelerated the regression of atheromatous lesions in the aorta as evidenced by significantly low sudanophilic staining. Andrea et al. (2002), shows positive effect of *A. caudatus *extract to decrease of cholesterol level, LDL-C, VLDL-C and TG [19]. Recent studies has shown *A. caudatus *extract decreased the most important risk factors of cardiovascular diseases the serum lipoproteins, apoB and Ox-LDL and inflammatory factors prevented atherosclerosis [20]. Amaranthus have Certain particularly soluble fibers, which have a high water holding capacity, appear to be effective in lowering the serum levels of total and LDL cholesterol in both normal and hyperlipidemic subjects [28]. Among the dietary interventions, dietary fibers play a crucial role in lipid lowering [29]. The effect of dietary fiber on cholesterol metabolism has been studied extensively. These effects are associated with increased excretion of bile acids and neutral sterols, increased catabolism of cholesterol, and reduced absorption of cholesterol and fat [30]. Squalene is an intermediate in cholesterol biosynthesis acts as a hyocholesterolemic agent by inhibiting HMG-CoA reductase, a necessary enzyme in cholesterol biosynthesis [17].

Atherosclerosis is widely viewed as an inflammatory disease [31]. Therefore, the reported inverse association between fruits and vegetable consumption(eight vs. two servings/day) and the selected proinflammatory gene expression and significantly reduced CRP levels [32,33]. The anti-inflammatory mechanisms related to fruit and vegetable consumption are still unclear. Fruits and vegetables also are important sources of dietary fiber [34], contain several flavonoids and carotenoids with recognized antioxidant properties which appears to have an anti-inflammatory role flavonoids and carotenoids, by an inhibition of NFκB activity, through suppressing the activation-related phosphorylation, and inhibiting the nuclear translocation [34,35]. Also, dietary fiber intake could participate in weight control and favor weight loss, hypoglycemic actions and hypolipidemic effects [36].

The flavanols and anthocyanins, and their role in the modulation or reduction of risk factors and the prevention of cardiovascular health problems through different aspects of bioeficacy in vascular health platelet agregation, atherosclerosis, blood pressure, antioxidant status, inflammation-related markers, *etc.*) are consistent. Anthocyanins seem to have a clear effect on endothelial function and myocardium protection [37], Anthocyanins have an effect on cholesterol distribution, protecting endothelial cells from CD40- induced proinflammatory signalling [38]. In macrophages, blackberry anthocyanins inhibit LPS induced nitric oxide biosynthesis [39]. Anthocyanins have anti inflammatory and free radical scavenging activity [39]. A great number of studies have shown that, anthocyanins prevents endothelial damages and act as an inhibitor of endothelial cell death [39,40]. Anthocyanins protect endothelial cell by inhibition peroxynitrites that it leads to oxidative damages [39]. The mechanism by which the Amaranthus reduces atheromatous lesion formation and accelerates the rate of lesion regression has not been examined in detail. The hypolipidemic action of the Amaranthus contributes to this. Although the mechanism of the antiinflammatory effect is not clear, the antiinflammatory effect as evidenced by a decrease in inflammatory markers such as CRP may also contribute to the protective effect stress oxidative.

## Conclusion

The objectives of the present investigation were *Amaranthus *produces regression of hypercholesterolemic atherosclerosis and regression is associated with reductions in serum lipids and oxidative stress. An investigation was therefore made of the effects of *Amaranthus *on the regression of atherosclerosis and serum lipids (cholesterol, triglyceride and LDL-cholesterol, HDL-C, ApolipoproteinB(apoB), apolipoproteinA(apoA), malondialdehyde (MDA), hs-C-reactive protein (hs-CRP) as well as atherosclerosis index (AI) and OX-LDL in rabbits. Oxidative stress parameters were assessed by measuring serum malondialdehyde (MDA) an index of levels of oxygen radicals, aortic antioxidant reserve and the oxygen radical-producing activity of white blood cells cells (WBC-CL). Our studies indicate that Amaranth can be considered as an effective natural antioxidant supplement capable of protecting cellular membranes against oxidative.

## Competing interests

The authors declare that they have no competing interests.

## Authors' contributions

NK Participated in the study design of study, interpretation of results, wrote the manuscript and proof read paper and made changes. SA designed and organized the research, contributed wrote the manuscript and proof read paper and made changes. MS contributed with the conception and design. All authors: read and approved the final manuscript.
